# Impaired Alveolar Barrier Integrity Exposes FGFR1 Orchestrating Tight Junction Homeostasis via Occludin Transcription Maintenance

**DOI:** 10.7150/ijbs.128209

**Published:** 2026-04-08

**Authors:** Jiaqi Zhang, Yang Zhao, Yujie Bao, Bo Yang, Qiaojun He, Zizheng Gao, Bing Xia, Peihua Luo

**Affiliations:** 1 Center for Drug Safety Evaluation and Research of Zhejiang University, College of Pharmaceutical Sciences, Zhejiang University, Hangzhou 310058, Zhejiang, P.R. China.; 2 School of Medicine, Hangzhou City University, Hangzhou 310015, P.R. China.; 3 Innovation Institute for Artificial Intelligence in Medicine of Zhejiang University, Hangzhou 310018, Zhejiang, P.R. China.; 4 Department of Thoracic Oncology, Hangzhou Cancer Hospital, Affiliated Hangzhou First People's Hospital, School of Medicine, Westlake University. Hangzhou 310006, Zhejiang, P.R. China.

## Abstract

The integrity of the alveolar epithelial barrier is fundamental to pulmonary homeostasis, yet the molecular regulators governing its tight junction (TJ) dynamics remain incompletely understood. Here, we uncover a critical, non-canonical function for FGFR1 as a master regulator of TJ homeostasis. We demonstrate that the RET inhibitor pralsetinib (Pral) induces fatal interstitial lung disease by inhibiting FGFR1, not RET, leading to the selective loss of occludin (OCLN). This disruption stems from transcriptional repression, mediated by the transcription factor CREB1. By integrating *in vitro* and *in vivo* studies, including lung epithelial-specific *Fgfr1*-knockout mice, we confirm that *Fgfr1* deficiency alone is sufficient to disrupt tight junctions, reduce OCLN expression, and trigger spontaneous lung injury. Our findings establish the FGFR1-CREB1-OCLN axis as a central regulator of TJ integrity, offering new therapeutic perspectives for lung diseases associated with TJ dysfunction.

## Introduction

Tight junctions (TJs) constitute barriers located at the apical region of cell-cell contacts 1. As evidenced by electron microscopy, TJs are described as sites where adjacent plasma membranes are closely apposed and exhibit partial fusion 2. TJ proteins in alveolar epithelial cells primarily consist of three components: claudins, OCLN, and zonula occludens (ZO) proteins. They are primarily responsible for maintaining the barrier function of the pulmonary epithelium and regulating the passage of fluid, ions, immune cells, etc. 3, 4. Extensive research has demonstrated that damage to pulmonary epithelial TJs is a significant contributor to various lung diseases. For instance, in acute respiratory distress syndrome (ARDS), FAS affects epithelial permeability by regulating TJ protein expression in alveolar epithelial cells, thereby promoting pulmonary edema 5. The tumor suppressor Pten influences the severity of pulmonary fibrosis by regulating lung epithelial integrity 6. Streptococcus pneumoniae promotes upper respiratory tract diseases by facilitating the degradation of OCLN in lung epithelial cells 7. Therefore, an in-depth investigation into the regulatory mechanisms of TJ protein expression in lung epithelial cells is pivotal for understanding pulmonary pathogenesis.

Fibroblast Growth Factor Receptor 1 (FGFR1) is a key member of the fibroblast growth factor receptor family and has been reported to orchestrate lung development, homeostasis, and regeneration 8, 9. Currently, the role of FGFR1 in pulmonary vascular endothelial cells has been relatively well characterized. Studies indicate that proper signaling through the FGFR1 pathway in endothelium mitigates smoke-induced chronic obstructive pulmonary disease (COPD) 10, alleviates vascular leakage and inflammation in acute respiratory distress syndrome (ARDS) 11, and promotes alveolar regeneration 12. Nevertheless, whether FGFR1 expression in alveolar epithelium elicits effects analogous to those in the endothelium, and whether FGFR1 blockade impairs pulmonary physiology by disrupting epithelial tight junctions, remains a fundamental unknown.

Herein, we focus on the role of the FGFR1 signaling pathway in the alveolar epithelium. Specifically, we discovered that the fatal interstitial lung disease (ILD) induced by the selective Rearranged during transfection (RET) inhibitor Pral (occurring in ~10% of cases, including 2.7% with Grade 3-4, and 0.5% with fatal reactions) 13 appears to depend on its effect on the expression of the epithelial TJ protein OCLN. Unexpectedly, this phenotype depended on the inhibition of FGFR1 rather than RET targeting 13. Further, we demonstrated that FGFR1 regulates OCLN expression by directing its transcription, and this process is dependent on the transcription factor CREB1. Critically, genetic knockdown (KD) or knockout (KO) *of Fgfr1* in lung epithelial cells led to dose-dependent reductions in OCLN protein, compromising pulmonary homeostasis even in the absence of external stimuli.

## Results

### Pral induces fatal ILD *in vivo*

**Figure 1:**
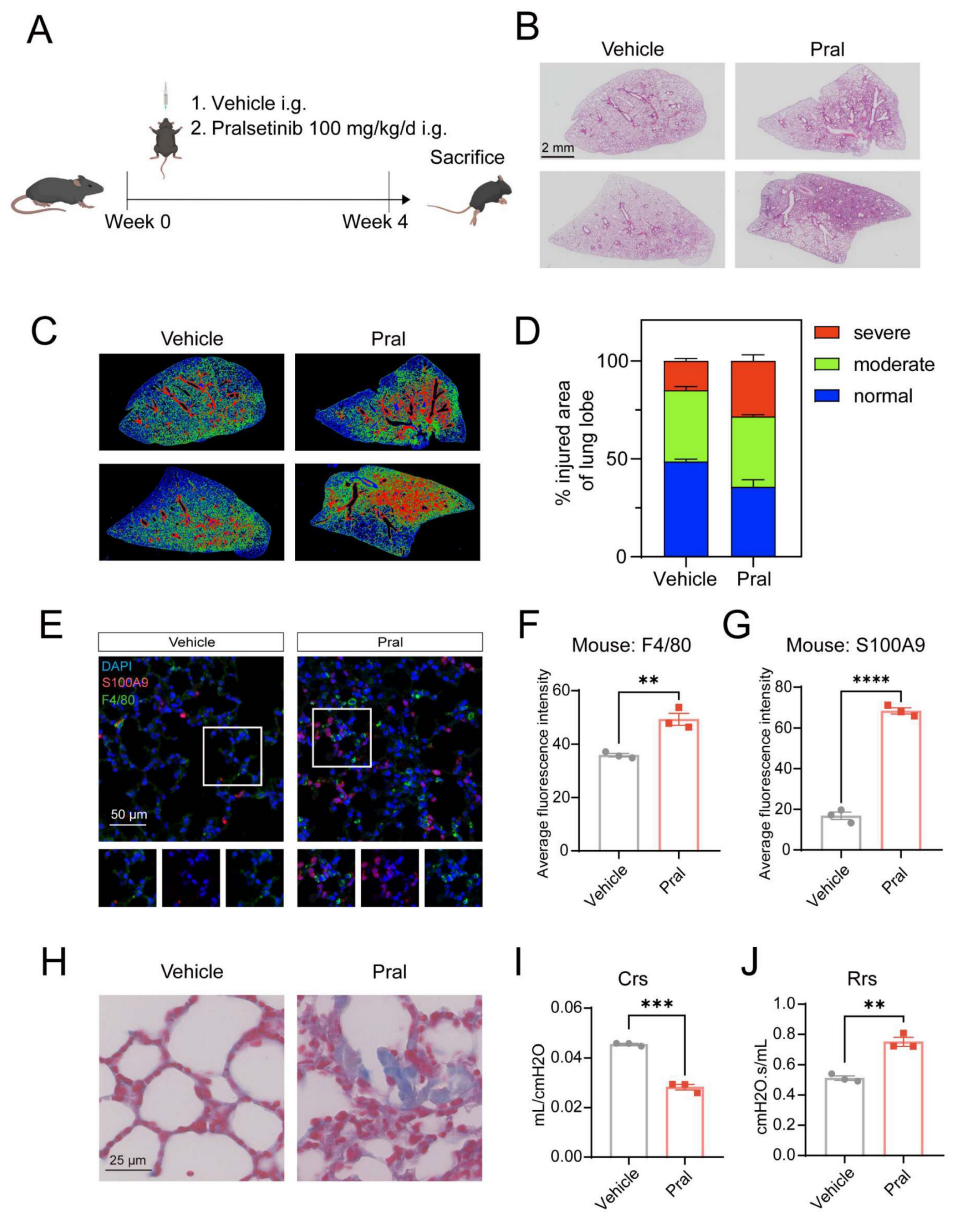
** Pral-associated fatal interstitial lung disease.** C57BL/6J mice were treated with either vehicle or 100 mg/kg/d Pral for four weeks (n=6). (A) Schematic of Pral administration. (B) Representative H&E-stained lung sections from Vehicle- and Pral-treated mice. (C) Injury-severity algorithm showing region of normal (blue), moderate injury (green), and severe injury (red). (D) Quantification of injury severity (% total lung area). (E-G) mIHC and quantification of immune cell infiltration:(E) Representative mIHC images showing F4/80^+^ macrophages (green) and S100A9^+^ neutrophils (red). (F) Average fluorescence intensity of F4/80. (G) Average fluorescence intensity of S100A9. (H) Representative images of Masson's trichrome staining highlighting collagen deposition. (I-J) Pulmonary function parameters: (I) Crs, (J) Rrs measured via the flexiVent system. Unpaired t-tests were used to assess significance between two groups. **: P < 0.01; ***: P < 0.001; ****: P < 0.0001.

To model Pral-induced fatal ILD *in vivo*, C57BL6/J mice were administered Pral at a dose of 100 mg/kg/d for four weeks. Lung tissues were subsequently collected for pathological analysis (Fig. 1A). Compared to a 100% survival rate in the vehicle group, the survival rate of Pral-treated mice decreased to approximately 50%, as shown in Fig. S1A. Log-rank (Mantel-Cox) test revealed a statistically significant difference between these two groups. Hematoxylin-eosin (H&E) staining showed extensive alveolar structural collapse and inflammatory cell infiltration in lung tissues after Pral administration, consistent with the pathological features of clinical ILD (Fig. 1B). Furthermore, employing the injury-severity algorithm described by Jones *et al*. 14, we demonstrated an increase in lung injury severity in Pral-treated mice (Fig. 1C, D). Additionally, multiplex immunohistochemistry (mIHC) revealed a significant increase in the positive rates of the macrophage marker F4/80 and the neutrophil marker S100A9 in lung tissues after treatment of Pral (Fig. 1E-G).

To further delineate the structural impact of Pral, we employed Masson's trichrome staining to evaluate fibrotic progression. Our results revealed that collagen deposition was significantly exacerbated in the Pral-treated group compared to the Vehicle group (Fig. 1H). Immunohistochemical (IHC) analysis further substantiated that Pral administration induced substantial pulmonary vascular remodeling (Fig. S1B). Moreover, to rigorously characterize the physiological impairment, we assessed murine pulmonary mechanics using the flexiVent system. The measurements of respiratory system compliance (Crs) and resistance (Rrs) indicated that Pral-treated mice developed diminished compliance and heightened resistance (Fig. 1I, J). Collectively, these findings confirm that Pral triggers a profound and fatal interstitial lung injury characterized by multifaceted structural and functional deterioration.

### Pral-triggered ILD depends on compromised tight junction integrity in lung epithelium

**Figure 2:**
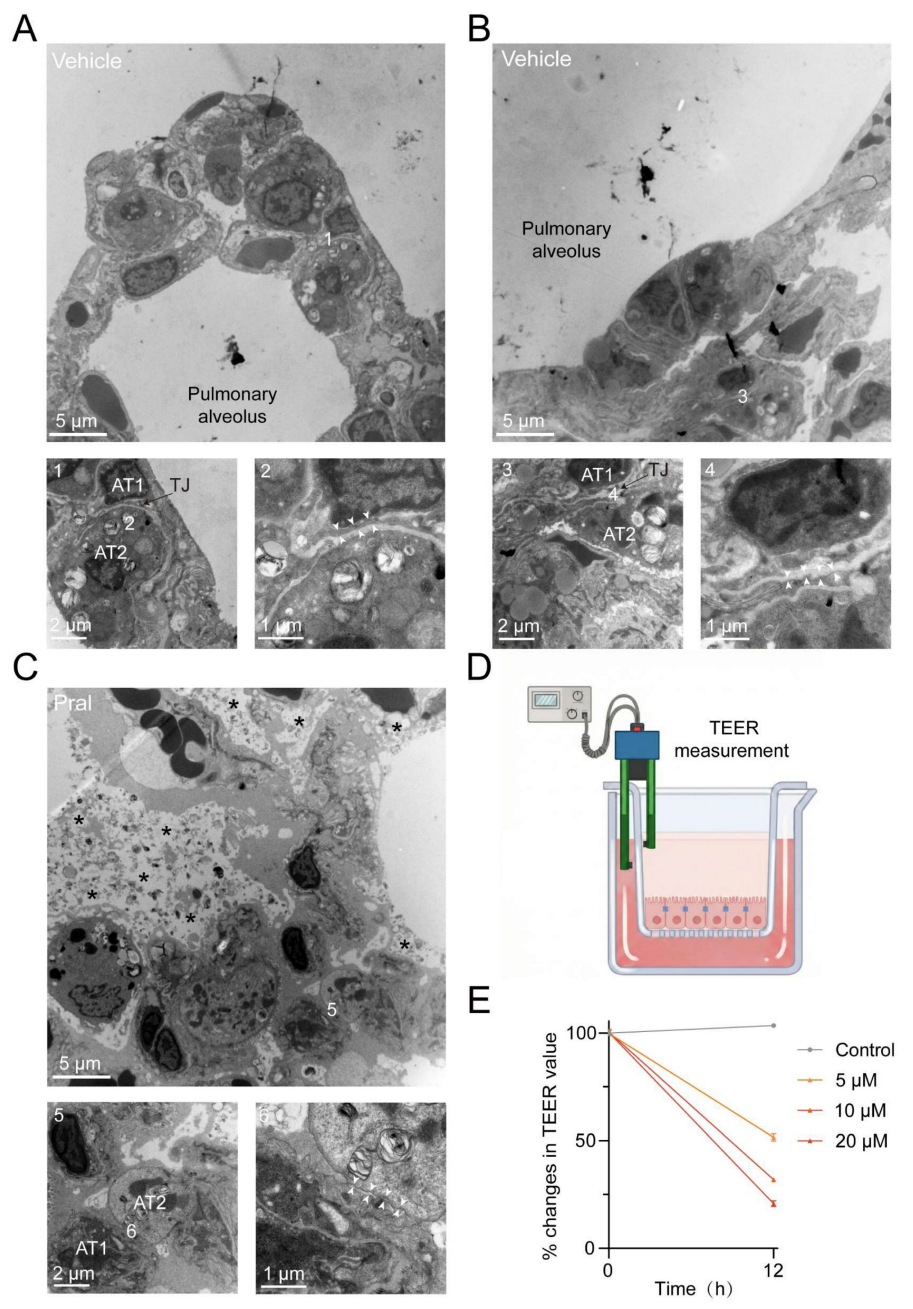
** Electron microscopy of Pral-triggered ultrastructural changes in the disrupted pulmonary epithelium TJ**. (A, B) Normal pulmonary epithelial cells. (C) Damaged pulmonary epithelial cells after Pral treatment. (A, B: 1-4, Intact tight junction structures; C: 5-6, Disrupted tight junction structures) AT1: Type I alveolar epithelial cell, AT2: Type II alveolar epithelial cell, White arrows indicate tight junctions between alveolar epithelial cells, asterisks indicate evident parenchymal edema. (D) Schematic diagram illustrating the experimental setup for TEER measurement. (E) Quantification of the percentage changes in TEER values in A549 cells treated with varying concentrations of Pral. Data are presented as mean ± SEM from three independent biological replicates.

To better investigate the cause of Pral-induced ILD, we used transmission electron microscopy (TEM) to examine the microstructural changes in the alveoli of the vehicle group and the Pral-treated group. Normal alveoli exhibited intact morphology and lacked inflammatory cell infiltration. Magnification revealed intact TJ structures between lung epithelial cells, collectively forming an impermeable barrier (Fig. 2A, B). In contrast, Pral-treated lung tissue showed collapsed alveolar structure, accompanied by extensive edema, deposition of collagen fibers, and inflammatory cell infiltration (Fig. S2A-B). Upon magnification, abnormal TJ structures were discovered between lung epithelial cells, indicating disruption of the pulmonary epithelial barrier function following Pral administration (Fig. 2C). To confirm the impairment of epithelial barrier function definitively, we measured the Trans-Epithelial Electrical Resistance (TEER) in Pral-treated A549 cells using a chopstick electrode system (Fig. 2D). The results demonstrated a dose-dependent decline in TEER values following Pral treatment, signifying an increase in barrier permeability (Fig. 2E). Given the critical role of TJ integrity in maintaining normal pulmonary physiological function, we proposed that Pral-induced TJ disruption in lung epithelium is a major cause of the fatal ILD.

### Epithelial OCLN repression drives Pral-induced tight junction disruption

**Figure 3:**
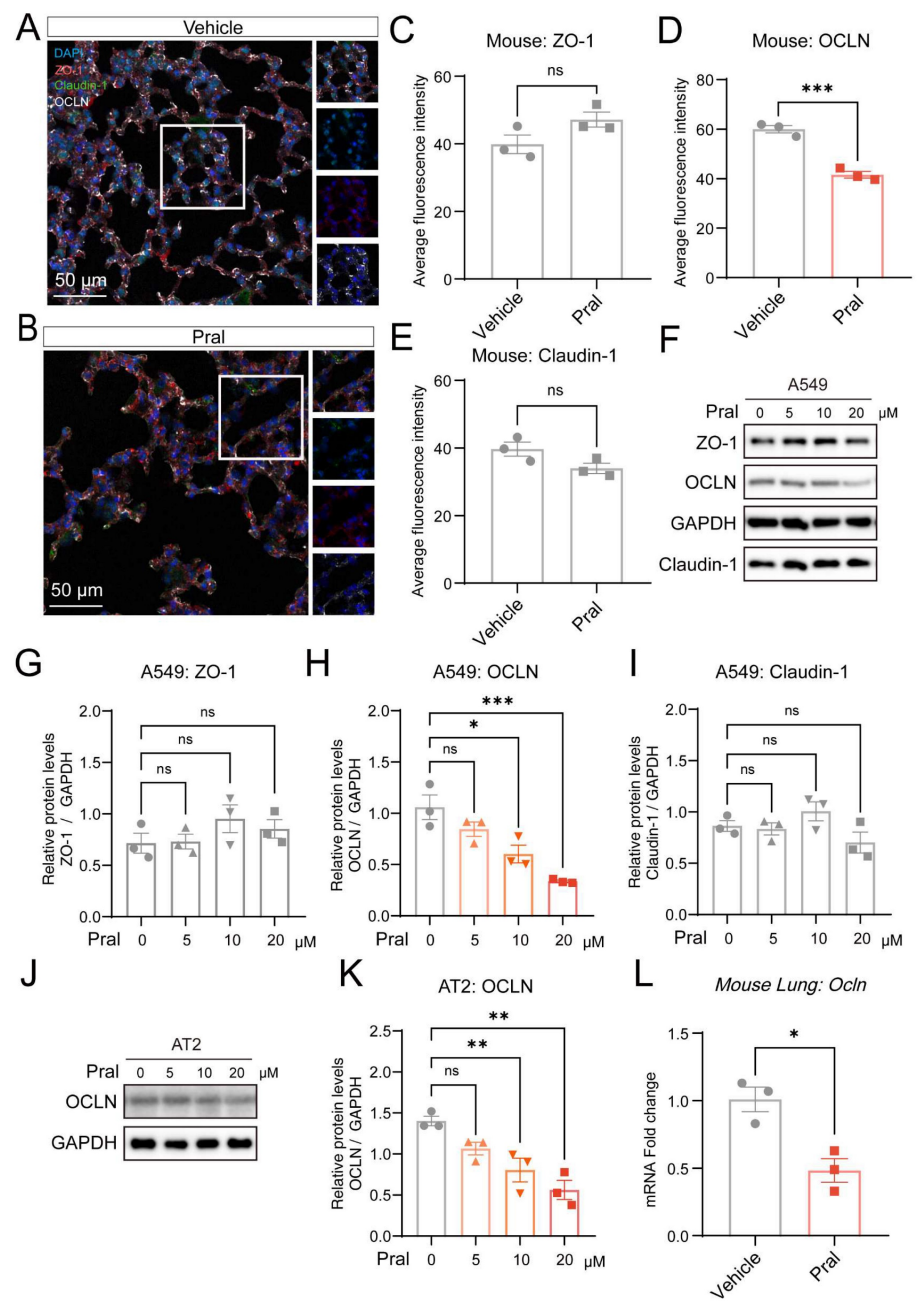
** Pral disrupts pulmonary epithelium TJ by suppressing OCLN expression.** (A-E) mIHC and quantification of pulmonary epithelium TJ. (A-B) Representative mIHC images of ZO-1 (red), OCLN (white), and Claudin-1 (green) in the Vehicle and the Pral group. (C-E) Quantification of ZO-1 (C), OCLN (D), and Claudin-1 (E) average fluorescence intensity. (F-I) Western blot analysis of TJ proteins in A549 cells. (F) Representative blots of ZO-1, OCLN, and Claudin-1 after Pral treatment. (G-I) Quantification of ZO-1 (G), OCLN (H), and Claudin-1 (I) protein levels. (J-K) Western blot analysis of OCLN in primary mouse AT2 cells. (J) Representative blots showing OCLN expression post-Pral treatment. (K) Quantification of OCLN protein levels. (L) qRT-PCR analysis of *Ocln* mRNA expression in mouse lung tissues following Pral treatment. All cell-based assays included three independent biological replicates. Statistical comparisons between two groups utilized unpaired two-tailed Student's t-tests, while multigroup analyses employed one-way ANOVA. Significance thresholds: ns (not significant), * (P < 0.05), ** (P < 0.01), *** (P < 0.001).

Further, we sought to investigate the mechanism by which Pral affects TJ in lung epithelial cells. As the TJ proteins of the pulmonary epithelial barrier primarily consist of ZO, OCLN, and Claudin proteins, we employed mIHC to examine their expression after Pral administration (Fig. 3A-B). We found no significant changes in the expression of ZO-1 and Claudin-1 proteins in lung tissues between the vehicle group and the Pral-treated group. However, the expression level of OCLN protein was significantly reduced after Pral administration (Fig. 3C-E). To determine whether Pral directly modulates OCLN within the pulmonary epithelium, we treated A549 and BEAS-2B cell lines with Pral and assessed the expression of ZO-1, Claudin-1, and OCLN via Western Blot (Fig. 3F; Fig. S3C). Consistent with our *in vivo* observations, OCLN protein expression exhibited a significant, dose-dependent decrease in both cell lines as drug concentrations increased. In contrast, no significant alterations were observed in the expression levels of other tight junction proteins (Fig. 3G-I; Fig. S3D-F). To further validate the impact of Pral on alveolar epithelial OCLN expression in a more physiologically relevant model, we treated primary mouse alveolar type II (AT2) cells. Our analysis confirmed that Pral treatment induced a reduction in OCLN expression in primary AT2 cells, mirroring the trends observed in both lung tissues and immortalized cell lines (Fig. 3J, K). To delineate the mechanism underlying Pral-induced OCLN suppression, we utilized qRT-PCR to detect OCLN expression in lung epithelial cells and mouse lung tissues following Pral administration. These results demonstrated that Pral triggers a significant decline in OCLN at the transcriptional level (Fig. 3L; Fig. S3G). In summary, Pral disrupts the TJs of the pulmonary epithelial barrier by impairing OCLN transcription in lung epithelial cells.

### FGFR1 inhibition drives Pral-induced OCLN downregulation

**Figure 4:**
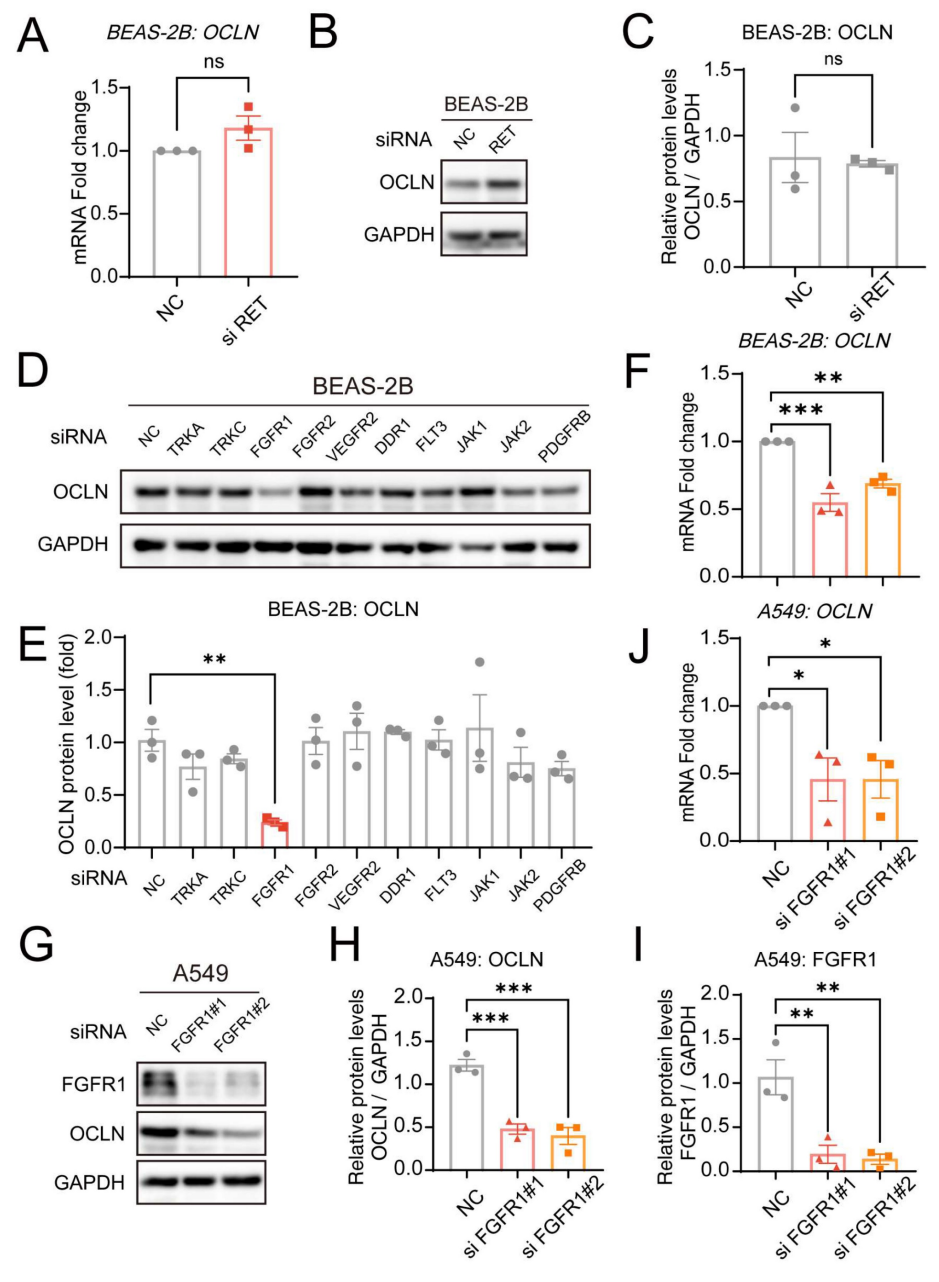
** Pral reduces OCLN expression through FGFR1 inhibition.** (A) qRT-PCR analysis of OCLN mRNA levels following *RET* silencing in BEAS-2B cells. (B-C) Western blot analysis of OCLN protein levels post-*RET* silencing in BEAS-2B cells. (B) Representative blots. (C) Quantification of OCLN. (D-E) Western blot analysis of OCLN protein expression after silencing other Pral targets in BEAS-2B cells. (D) Representative blots. (E) Quantification of OCLN. (F) qRT-PCR analysis of OCLN mRNA levels post-*FGFR1* silencing in BEAS-2B cells. (G-I) Western blot analysis of OCLN protein levels post-*FGFR1* silencing in A549 cells. (G) Representative blots. (H-I) Quantification of OCLN. (I)* FGFR1* knockdown efficiency. (J) qRT-PCR analysis of OCLN mRNA levels following *FGFR1* silencing in A549 cells. Statistical comparisons between two groups utilized unpaired two-tailed Student's t-tests, while multigroup analyses employed one-way ANOVA. Significance thresholds: ns (not significant), * (P < 0.05), ** (P < 0.01), *** (P < 0.001).

To delineate how Pral suppresses OCLN expression, we initially focused on its direct inhibition targets. As a selective RET inhibitor, Pral has potent inhibitory activity against the RET tyrosine kinase. Therefore, we initially hypothesized that Pral-induced downregulation of OCLN expression might depend on RET inhibition. Consequently, we silenced RET in BEAS-2B cells using siRNA, and silencing efficiency was confirmed via qRT-PCR (Fig. S4A). However, the transcriptional and protein levels showed no significant effect on OCLN expression after the RET gene had been silenced (Fig. 4A-C). Next, referring to the FDA approval summary for Pral, besides RET inhibition, Pral also inhibits DDR1, TRKC, FLT3, JAK1-2, TRKA, VEGFR2, PDGFRb, and FGFR1-2 at clinically achievable doses 13. Therefore, we silenced each of these genes using siRNA, and Western Blot was used to assess the impact on OCLN expression (Fig. 4D). Among them, only inhibition of FGFR1 resulted in a significant decrease in OCLN expression in lung epithelial cells, while others showed no significant change (Fig. 4E). Currently, the ability of FGFR1 to influence OCLN has been characterized to some extent in Blood-brain barrier (BBB) research 15. However, FGFR1-dependent regulation of OCLN within pulmonary epithelial barriers is unexplored. Subsequently, we used qRT-PCR to detect the effect of *FGFR1* silencing on OCLN transcription, finding a significant decrease compared to the vehicle group, consistent with the effect of Pral treatment (Fig. 4F, Fig. S4B). To further validate FGFR1 inhibition-mediated OCLN downregulation in lung epithelia, we employed a second pulmonary epithelial cell line (A549). qRT-PCR and Western blot analyses confirmed that *FGFR1* silencing in A549 cells reduces OCLN expression, mirroring observations in BEAS-2B cells (Fig. 4G-J, Fig. S4C). Thus, we established that *FGFR1* silencing mediates Pral-induced OCLN downregulation.

### FGFR1 regulates OCLN expression levels via the transcription factor CREB1

**Figure 5:**
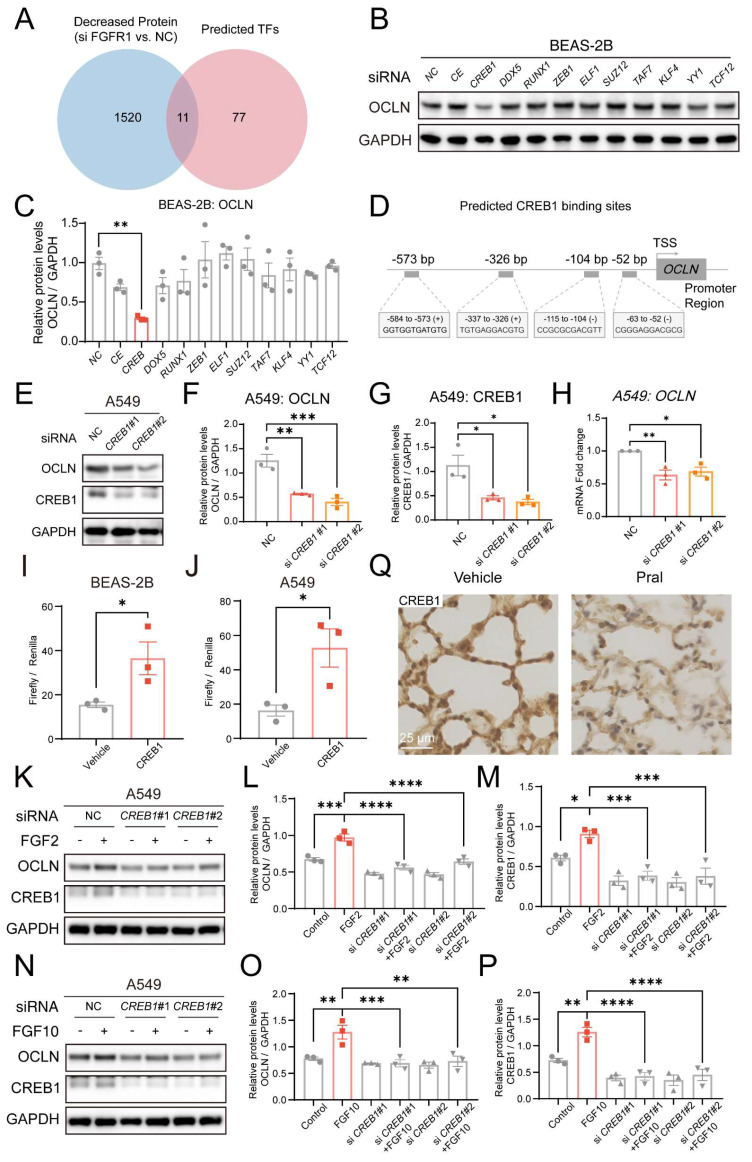
** FGFR1 inhibition reduces OCLN transcription via CREB1 suppression.** (A) Venn diagram intersecting proteins downregulated (≥1.5-fold) after *FGFR1* silencing in lung epithelial cells with OCLN transcription factors predicted by the hTFtarget database. (B-C) Western blot analysis of OCLN expression in BEAS-2B cells following silencing of candidate transcription factors. (B) Representative blots. (C) Quantification of OCLN protein levels. (D) JASPAR database prediction of CREB1 binding sites in the OCLN promoter region. (E-G) Western blot analysis of OCLN protein in CREB1-silenced A549 cells. (E) Representative blots. (F) Quantification of OCLN levels. (G) CREB1 knockdown efficiency. (H) qRT-PCR analysis of OCLN mRNA in CREB1-silenced A549 cells. (I-J) Luciferase reporter assay validating CREB1 binding to the *OCLN* promoter in BEAS-2B (I) and A549 (J) cells. Activity is expressed as the ratio of Firefly to Renilla luciferase. (K-M) Western blot analysis of the FGFR1-CREB1-OCLN axis in A549 cells stimulated with FGF2 (+/- si-*CREB1*). (K) Representative blots; (L-M) Quantification of OCLN and CREB1 protein levels. (N-P) Western blot analysis of the FGFR1-CREB1-OCLN axis in A549 cells stimulated with FGF10 (+/- si*-CREB1*). (N) Representative blots; (O-P) Quantification of OCLN and CREB1 protein levels. (Q) IHC staining of CREB1 protein in mouse lungs post-Pral treatment. Statistical comparisons between two groups utilized unpaired two-tailed Student's t-tests, while multigroup analyses employed one-way ANOVA. Significance thresholds: ns (not significant), * (P < 0.05), ** (P < 0.01), *** (P < 0.001), **** (P < 0.0001).

Since FGFR1 regulates OCLN expression levels in a transcription-dependent manner, we hypothesized that FGFR1 might influence OCLN expression by regulating downstream transcription factors. To test this hypothesis, we first screened the hTFtarget database for predicted OCLN transcription factors, followed by joint analysis with proteins downregulated (≥1.5-fold) after *FGFR1* had been silenced (Fig. 5A). This analysis identified 11 overlapping transcription factors: CEBPB, CREB1, DDX5, RUNX1, ZEB1, ELF1, SUZ12, TAF7, KLF4, YY1, and TCF12. Western Blot analysis demonstrated that only CREB1 silencing significantly reduced OCLN expression, whereas other genes failed to alter OCLN levels (Fig. 5B, C). Notably, the JASPAR database revealed a significant enrichment of CREB1 binding sites within the OCLN promoter region, indicating direct transcriptional regulation of OCLN by CREB1 (Fig. 5D, Fig. S5A). To validate this, we silenced CREB1 in lung epithelial cells and confirmed its regulatory effect on OCLN through Western Blot and qRT-PCR, which established parallel OCLN suppression to FGFR1 inhibition (Fig. 5E-H, Fig.S5B). Additionally, CREB1 expression was also reduced after *FGFR1* silencing, consistent with the omics results (Fig. S5C-H). To further confirm the regulatory role of CREB1 as a transcription factor for OCLN, we constructed luciferase reporter plasmids by fusing the *OCLN* promoter region to a luciferase-expressing fragment. Overexpression of CREB1 in lung epithelial cells resulted in a marked upregulation of luciferase activity, establishing the direct binding capacity of CREB1 to the *OCLN* promoter (Fig. 5I, J).

Furthermore, to rigorously validate the integrity of the FGFR1-CREB1-OCLN signaling axis, we performed rescue experiments using exogenous FGF stimulation. Lung epithelial cells were stimulated with FGF2 or FGF10, well-recognized ligands for FGFR1, following *CREB1* knockdown. Western blot analysis demonstrated that the silencing of *CREB1* effectively abolished the FGF-mediated upregulation of OCLN (Fig. 5K-P; Fig. S5I-N). Additionally, we observed significant CREB1 reduction in lung tissues and epithelial cells following Pral treatment (Fig. 5Q; Fig. S5O, P). Collectively, these data concluded that FGFR1 regulates OCLN expression in lung epithelial cells through CREB1.

### Epithelial-specific *Fgfr1* deletion recapitulates barrier disruption and lung injury via the CREB1-OCLN axis

**Figure 6:**
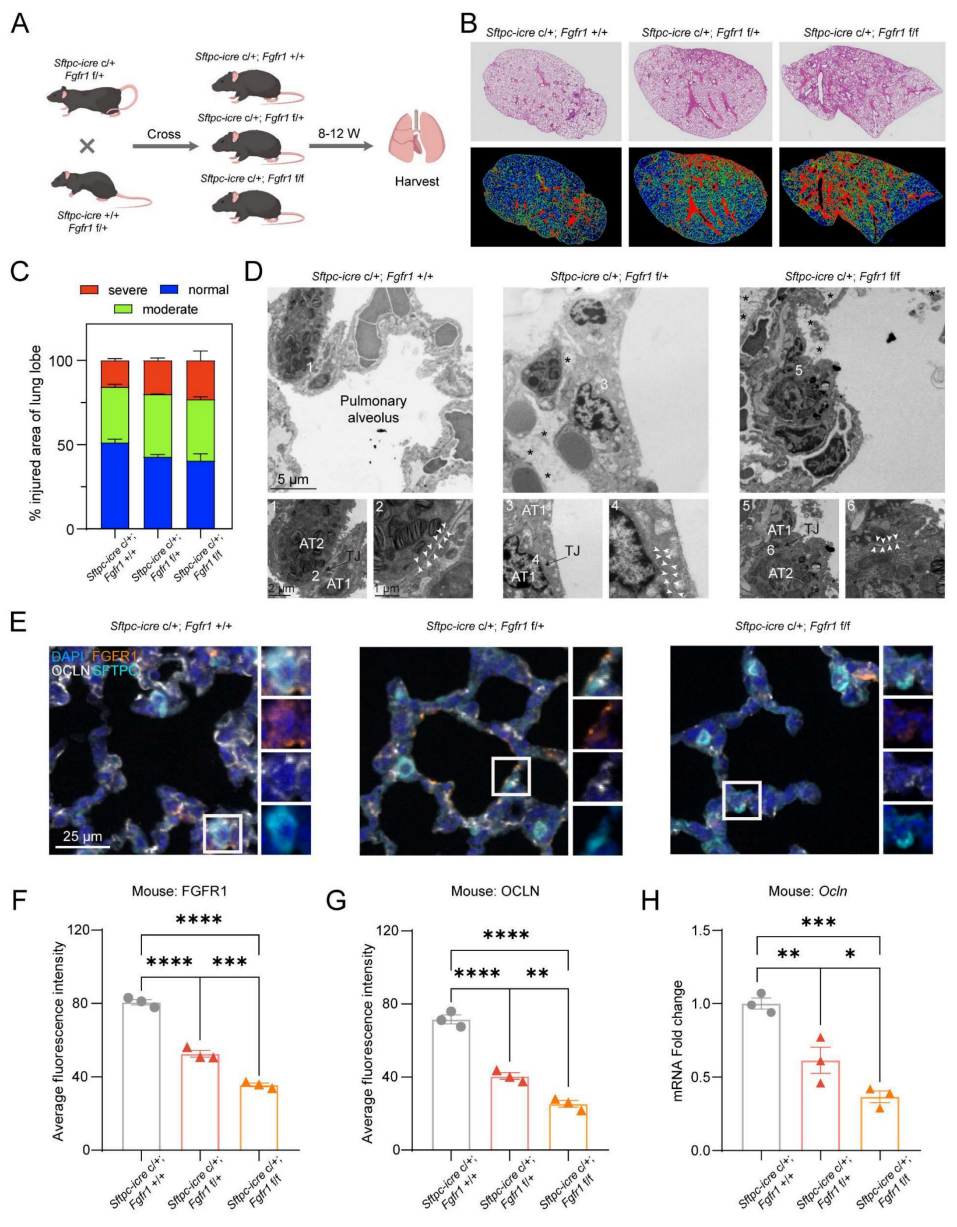
** Lung epithelial-specific *Fgfr1* knockdown/knockout triggers OCLN downregulation and interstitial lung disease.** (A) Schematic of the genetic cross strategy used to generate lung epithelial-specific *Fgfr1* KD/KO mice (created with biogdp.com). (B) Representative H&E-stained lung sections from control and *Fgfr1* KD/KO mice. (C) Injury-severity mapping of lung sections: normal (blue), mild injury (green), and severe injury (red). (D) Transmission electron microscopy images of pulmonary epithelial tight junctions in control and *Fgfr1* KD/KO mice. AT1: type I alveolar epithelial cell; AT2: type II alveolar epithelial cell. White arrows indicate tight junctions; asterisks indicate parenchymal edema. (E-G) mIHC and quantification of FGFR1 and OCLN expression. (E) Representative mIHC images of FGFR1 (orange), SFTPC (cyan), and OCLN (white) in lung tissues from control and *Fgfr1* KD/KO mice. (F-G) Quantification of average fluorescence intensity for FGFR1 (F) and OCLN (G). (H) qRT-PCR analysis of *OCLN* mRNA levels in lung tissues from control and *Fgfr1* KD/KO mice. Statistical comparisons between multigroup analyses employed one-way ANOVA. Significance thresholds: ns (not significant), * (P < 0.05), ** (P < 0.01), *** (P < 0.001), **** (P < 0.0001).

To further validate the role of FGFR1 in maintaining the pulmonary epithelial barrier, we crossed (*Sftpc-icre* c/+; *Fgfr1* f/+) mice with (*Sftpc-icre* +/+; *Fgfr1* f/+) mice to generate mice with lung epithelial-specific KD/KO of FGFR1. The (*Sftpc-icre* c/+; *Fgfr1* +/+) mice littermates were used as controls (Fig. 6A, Fig. S6A). H&E staining revealed that the extent of lung injury significantly increased in correlation with the degree of *Fgfr1* KO, demonstrating a clear dose-dependent relationship (Fig. 6B, C). Furthermore, mIHC results confirmed that KO of *Fgfr1* led to increased infiltration of macrophages and neutrophils, a phenotype consistent with the pulmonary alterations observed following Pral administration (Fig. S6B-D). To directly assess the impact of FGFR1 on tight junctions, we examined the pulmonary ultrastructure of the three genotypes using TEM. The results indicated that KO of *Fgfr1* resulted in disruption of pulmonary epithelial tight junctions, whereas control mice exhibited intact tight junction structures (Fig. 6D). Subsequently, mIHC co-staining for FGFR1 and OCLN demonstrated that *Fgfr1* KO mice exhibited significantly reduced expression of both FGFR1 and OCLN in lung tissue, further supporting the essential regulatory role of FGFR1 in regulating OCLN expression (Fig. 6E-G). This finding was corroborated by qRT-PCR, which showed a pronounced downregulation of OCLN transcription following *Fgfr1* deletion (Fig. 6H). Additionally, IHC analysis of CREB1 revealed that KO of *Fgfr1* correspondingly reduced its expression (Fig. S6E-F). Collectively, these data substantiate the critical role of the FGFR1-CREB1-OCLN signaling axis in regulating tight junction integrity and homeostasis in pulmonary epithelial cells.

## Discussion

**Figure 7:**
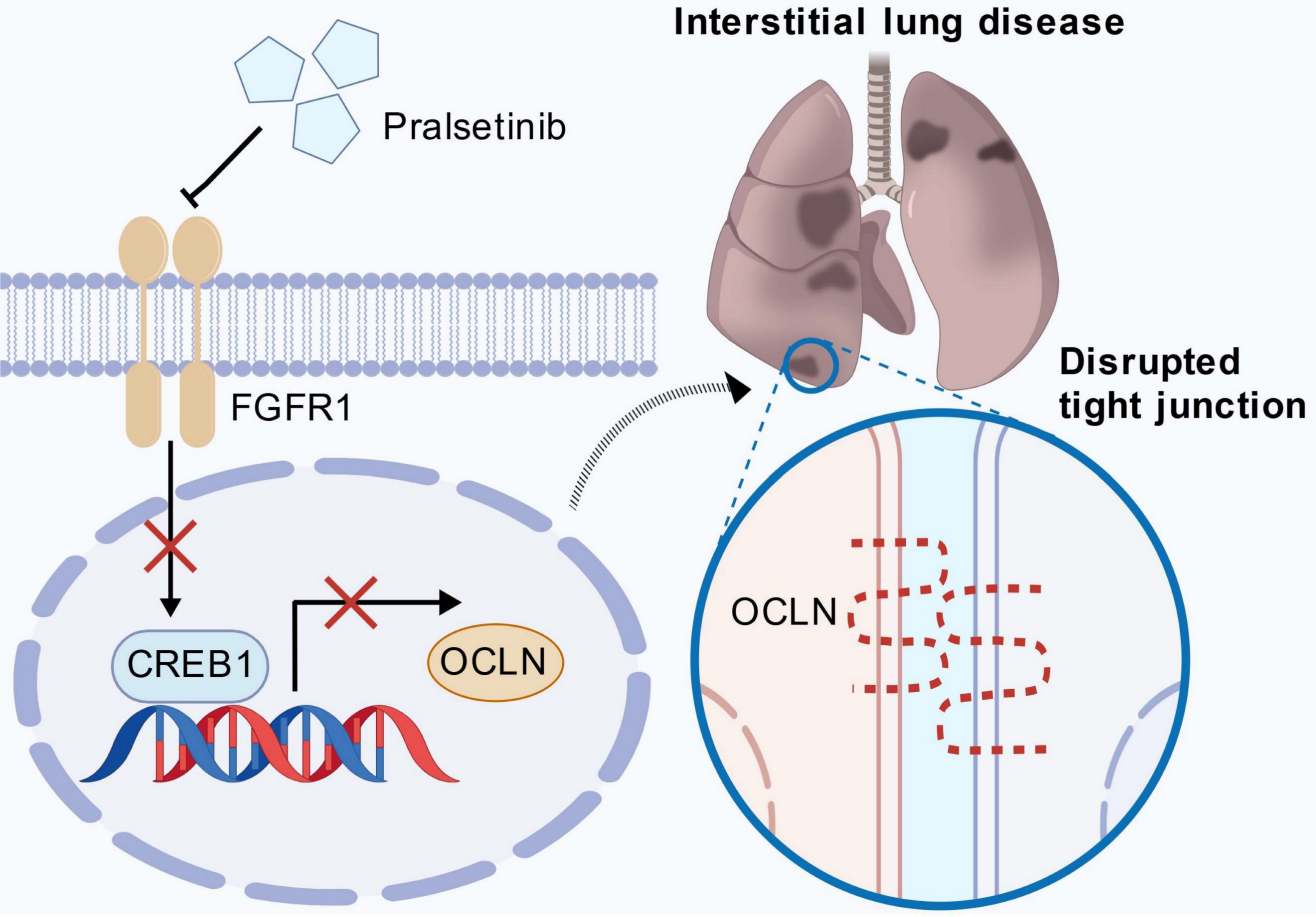
** FGFR1 maintains pulmonary epithelial barrier integrity through the CREB1-OCLN transcriptional axis.** Under homeostatic conditions, active FGFR1 signaling promotes the transcription of OCLN through the transcription factor CREB1. Genetic knockdown or pharmacological inhibition of FGFR1 (e.g., by Pral) disrupts this axis, leading to a sharp decline in CREB1 expression. Consequently, *OCLN* transcription is profoundly suppressed, leading to the disassembly of tight junctions and compromising the epithelial barrier. This breach allows for fluid influx and inflammatory cell infiltration, ultimately culminating in alveolar damage and the pathogenesis of ILD (created with biogdp.com).

While FGFR1 is canonically linked to angiogenesis and developmental patterning 16, we identify its non-canonical role as a master regulator of alveolar barrier integrity through direct transcriptional control of OCLN (Fig. 7). Genetic or pharmacological FGFR1 inhibition disrupts tight junction homeostasis by suppressing OCLN expression, with CREB1 serving as the essential downstream transcriptional effector that binds the OCLN promoter. This FGFR1-CREB1 axis orchestrates sustained OCLN synthesis, which enriches the prevailing model that FGFR1 primarily safeguards vascular homeostasis, establishing FGFR1 as a gatekeeper of pulmonary epithelial stability.

Our findings elucidate the etiology of Pral-induced interstitial lung disease (ILD): FGFR1 inhibition disrupts the CREB1-OCLN axis, provoking barrier failure without RET dependency. This suggests FGFR1 occupancy as an underappreciated determinant of TKI safety profiles. Monitoring CREB1 or alveolar OCLN levels could stratify ILD risk, while engineered FGFR1-sparing RET inhibitors may mitigate this lethal adverse effect.

Despite the novel mechanistic insights provided by this study, several limitations warrant further consideration. First, while our investigation primarily focused on the pivotal role of OCLN in Pral-induced ILD, it is plausible that other tight junction proteins also contribute to the observed barrier dysfunction. Consistent with previous reports suggesting the co-regulation of Claudin-18 and OCLN in the lung parenchyma 17, our supplementary data revealed a significant downregulation of Claudin-18 in both lung epithelial cells and lung tissues following Pral administration (Fig. S7A-C). This observation suggests that Pral-induced disruption of the epithelial barrier likely involves a broader suppression of the tight junction complex beyond OCLN alone. Second, although we demonstrated impaired barrier integrity via TEER measurements and ultrastructural analysis, this study lacks a direct, real-time assessment of alveolar epithelial permeability *in vivo*. This limitation restricts our ability to precisely quantify the magnitude of epithelial-specific leakage during the early stages of Pral-induced injury.

Beyond TKIs, the FGFR1-CREB1-OCLN axis likely underpins barrier pathologies in diverse contexts. Given the damage to the tight junction barrier of lung epithelium in various lung diseases, we posit this pathway as a fundamental modulator for pulmonary epithelial barrier resilience, with therapeutic potential extending to ARDS or pulmonary fibrosis. Furthermore, investigating whether FGFR1-overexpressing transgenic mice exhibit reduced susceptibility to lung insults represents a compelling research direction to further validate the protective capacity of this signaling axis. Future studies should map ligand specificity (e.g., FGF2 vs. FGF10) and explore CREB1 co-regulators that fine-tune OCLN transcription in stress responses.

## Conclusion

Our study establishes FGFR1 as a pivotal guardian of pulmonary epithelial barrier integrity through CREB1-mediated transcriptional control of OCLN. Genetic or pharmacological inhibition of FGFR1 disrupts this axis, triggering tight junction disassembly and spontaneous interstitial lung disease - independent of RET signaling. This reveals a tissue-specific vulnerability in alveolar epithelium and positions FGFR1 targeting as a therapeutic strategy for barrier dysfunction in diverse pulmonary pathologies.

## Supplementary Material

Supplementary methods and figures.
