# Supplementary material for: Impaired Alveolar Barrier Integrity Exposes FGFR1 Orchestrating Tight Junction Homeostasis via Occludin Transcription Maintenance
Source: Int J Biol Sci. 2026 Apr 8;22(8):4334–45. doi: 10.7150/ijbs.128209 (PMC13137958; doi:10.7150/ijbs.128209)
Supplement: Supplementary file 1 — Supplementary methods and figures. [file ijbsv22p4334s1.pdf]

Materials and Methods

Animal Study and Approval

C57BL/6J mice (male, 8 weeks) were sourced from Vital River Laboratory Animal Technology (Jiaxing, China) and maintained under specific pathogen-free conditions with controlled 12-hour light/dark cycles. Animals received ad libitum access to autoclaved feed and sterile water. All experimental protocols were approved by the Center for Drug Safety Evaluation and Research of Zhejiang University and performed according to the Institutional Animal Care and Use Committee (IACUC) protocol of Zhejiang University (ethical approval: IACUC-s20-025). Following one week of acclimation, drug administration commenced. Terminal procedures involved anesthesia induction with 2% isoflurane followed by cervical dislocation for tissue collection.

Pral (TQ0277, TargetMol, USA) was solubilized in cyclodextrin (Aladdin, H108813) to generate stock solutions. Seven- to ten-week-old mice received daily intragastric gavage of either vehicle control (cyclodextrin) or 100 mg/kg/d Pral for 28 consecutive days.

Histopathological and Immunohistochemical Analysis

Post-euthanasia lung specimens were divided into two parts. The right part underwent snap-freezing in liquid N_2 for storage at -80 °C. The left lobe was immersion-fixed in 4% paraformaldehyde, embedded in paraffin, and sectioned at 4 μm. Tissue sections were stained with H&E and Masson's trichrome staining per standardized protocols. Pulmonary injury quantification employed the injury-severity algorithm described by Jones et al [14].

For IHC, sections were incubated with anti-CREB1 (ET1601-15, HuaBio, China), with subsequent whole-slide digital scanning and analysis (HS6, Sunny Instrument Co., Ltd., Ningbo, China).

Multiplex immunohistochemistry (mIHC) was performed by Hunan Aifang Biological following standardized protocols. Primary antibodies utilized included: F4/80 (29414-1-AP, Wuhan Sanying, China), S100A9 (29414-1-AP, Wuhan Sanying, China), FGFR1 (ET1612-62, HuaBio, China), OCLN (27260-1-AP, Wuhan Sanying, China), SFTPC(10774-1-AP, Wuhan Sanying, China).

Pulmonary Function Measurement

To characterize the physiological consequences of Pral on lung mechanics, we evaluated respiratory function using a flexiVent apparatus (SCIREQ, Montreal, Canada). Following the induction of deep anesthesia, mice were positioned supine and firmly stabilized for consistent data acquisition. Tracheal exposure was achieved via a midline cervical incision, allowing for the precise insertion of an endotracheal cannula. This cannula was subsequently interfaced with the ventilator, and key parameters—including Crs and Rrs—were recorded and processed using the integrated FlexiWare software.

Cell Culture and Drug Treatment

Sourced from Jennio Biotech (Guangzhou, China) and the Typical Culture Collection Committee of the Chinese Academy of Sciences (Shanghai, China), respectively, BEAS-2B and A549 cell lines were used in this study. BEAS-2B cells were grown in DMEM (12800, Gibco, Carlsbad, USA), while A549 cells were cultured in RPMI-1640 (31800, Gibco, Carlsbad, USA), both of which were supplemented with 10% FBS (Hyclone) and a standard antibiotic mixture (100 U/mL penicillin and 100 μg/mL

streptomycin). Incubation was carried out under standard conditions of 37°C and 5% CO_2.

As for primary mouse alveolar type II (AT2) cells, the isolation and extraction of these primary cells were conducted by Zhejiang Meisen Cell Technology Co., Ltd. (Hangzhou, China). Briefly, the lung vasculature was perfused with sterile PBS via the right ventricle to clear residual blood cells. The lungs were then inflated with dispase, followed by low-melting-point agarose. The lung tissue was subsequently dissociated into a single-cell suspension through mechanical mincing and enzymatic digestion. Primary AT2 cells were purified using a negative selection strategy involving magnetic bead depletion of CD45+ leukocytes and CD31+ endothelial cells, followed by differential adherence to remove fibroblasts.

The purified primary AT2 cells were cultured in specialized Mouse Primary Alveolar Type II Epithelial Cell Medium (CTCC-A005-MIC-CM, Meisen Cell, Hangzhou, China). The medium was supplemented with 100 U/mL penicillin and 100 μg/mL streptomycin to prevent microbial contamination. Cells were maintained in a humidified atmosphere at 37°C with 5% CO2.

All cell lines tested negative for mycoplasma contamination. Cells were treated with 5, 10, or 20 μM Pral for qRT-PCR and Western blot analyses. In specified conditions, cells were co-treated with 20 ng/mL FGF2 (HY-P70440, MedChemExpress, USA) and 50 ng/mL FGF10 (HY-P7048, MedChemExpress, USA).

TEER Measurement

For the in vitro assessment of alveolar barrier robustness, A549 cells were plated at an initial density of 5×10^3 cells per well into 24-well Transwell inserts (0.4 μm pore size; BIOFIL, China). After the specified experimental interventions, we quantified the

TEER. These measurements were conducted using an RE1600 epithelial volt-ohm meter sourced from Beijing Jingong Hongtai (China).

Dual-Luciferase Reporter Assay

To evaluate transcriptional activity, we employed a dual-luciferase reporter system where Firefly luciferase acted as the primary reporter and Renilla luciferase served as an internal control for data normalization. The CREB1 overexpression and OCLN promoter-Luciferase plasmids were custom-synthesized by Wuhan MiaoLingBio (Wuhan, China). The experimental procedure began with the transfection of the CREB1 expression vector, followed by a medium refresh after 5 hours. Subsequently, the OCLN promoter-Luciferase and Renilla constructs were introduced into cells at a 10:1 ratio, with a second medium replacement performed 5 hours later. Luminescence was quantified by first equilibrating the culture plates to room temperature (30 min). Using the Duo-Lite Luciferase Assay System (Vazyme Biotech, Nanjing, China), Firefly activity was measured after a 10-minute incubation with the detection reagent. This was followed by the addition of Stop & Lite reagent to measure Renilla activity (10-minute incubation). Transcriptional potential was determined by calculating the Firefly/Renilla activity ratio.

Proteomics

Proteomic profiling was conducted by Shanghai OE Biotech Co., Ltd. (Shanghai, China). Samples were analyzed using a Thermo Scientific Vanquish Neo UHPLC system coupled to an Orbitrap Astral mass spectrometer equipped with an Easy-spray ion source. The study design included a negative control (NC) group and an *FGFR1*-silenced (si*FGFR1*) group. Key procedural steps encompassed: protein extraction,

SDS-polyacrylamide gel electrophoresis (SDS-PAGE), in-solution digestion, liquid chromatography-tandem mass spectrometry (LC-MS/MS), and database searching.

Transmission electron microscopy analysis

For the assessment of pulmonary ultrastructure, lung specimens were obtained from the experimental mice and subjected to initial fixation in 1 mL of fresh 2.5% glutaraldehyde (PH9003, Scientific Phygene) for 2 hours at ambient temperature, prior to 4°C refrigeration overnight. Tissues then underwent a 1-hour secondary fixation in 1% osmium tetroxide, which was succeeded by a 30-minute en bloc staining process with 2% uranyl acetate. Subsequent to graded dehydration and embedding in epoxy resin, a diamond knife was utilized to prepare ultrathin sections. Final visualization and morphological image acquisition were achieved using a TECNAI 10 transmission electron microscope (Thermo Fisher Scientific).

Immunofluorescence Assay

Following treatment, cultured cells in 96-well plates underwent two PBS washes, followed by fixation with 4% paraformaldehyde (P6148, Sigma-Aldrich, USA) for 20 minutes and permeabilization with 0.1% Triton X-100 in PBS for 10 minutes at 4°C. Subsequent blocking utilized 4% bovine serum albumin (B2064, Sigma-Aldrich, USA) in PBS at 37°C for 30 minutes, after which primary antibody incubation proceeded overnight at 4°C. Following additional PBS washes, cells were exposed to Alexa Fluor 594-conjugated secondary antibodies (A21206; 1:250, Thermo Fisher Scientific, USA) for 1 hour at room temperature. Nuclear counterstaining was performed using DAPI (D212, Dojindo, Japan) for 5 minutes, with fluorescence imaging conducted on a Leica TCS SP8 microscope.

Western Blot

Protein lysates were prepared from cellular samples using a lysis buffer formulation containing 150 mM NaCl, 50 mM Tris-HCl, 2 mM EGTA, 2 mM EDTA, 25 mM β-glycerophosphate, 25 mM NaF, 0.3% Triton X-100, 0.3% NP-40, 0.3% leupeptin, 0.1% $NaVO_3$, and 0.1% PMSF. Aliquots of 30-50 μg protein were electrophoretically resolved on 8%, 10%, or 12% SDS-polyacrylamide gels and transferred to 0.45 μm PVDF membranes (IPVH00010, Millipore). Membranes were blocked with non-fat milk prior to sequential incubation with primary and secondary antibodies, with protein detection achieved via ECL Plus chemiluminescent substrate (P2300, NCM Biotech). Primary immunoreagents included: FGFR1 (ET1612-62, HuaBio, China), CREB1 (ET1601-15, HuaBio, China), OCLN (91131, CST, USA), ZO-1 (HA722797, HuaBio, China), Claudin-1 (HA721248, HuaBio, China), GAPDH (HA721136, HuaBio, China)

Quantitative real-time PCR (qPCR)

Following specified treatments, cellular and murine tissue samples underwent RNA extraction using TRIzol reagent (15596026, Thermo Scientific, USA). Equivalent RNA quantities were reverse transcribed into cDNA with a cDNA synthesis kit (AT311, TransGen Biotech, China). Quantitative real-time PCR amplification was conducted using TB Green Premix Ex Taq (Tli RNaseH Plus, RR420A, Takara, Japan) on a QuantStudio 3 system (A28132, Applied Biosystems, Singapore). Gene expression fold changes were derived via the $2^{-\Delta\Delta Ct}$ method through comparative analysis of threshold cycle (Ct) values. Dual biological replicates were analyzed in technical duplicates, with expression data normalized to reference genes. Primer sequences were as follows:

OCLN forward: GCAAAGTGAATGACAAGCGG

OCLN reverse: CACAGGCGAAGTTAATGGAAG

FGFR1 forward: AACCTGCCTTATGTCCAGATC

FGFR1 reverse: AGAGTCCGATAGAGTTACCCG

RET forward: GGAGAAGGCGAATTTGGAAAAG

RET reverse: CAGGACGTTGAACTCTGACAG

Ocln forward: ACTATGCGGAAAGAGTTGACAG

Ocln reverse: GTCATCCACACTCAAGGTCAG

Fgfr1 forward: AACTTGCCGTATGTCCAGATC

Fgfr1 reverse: AGAGTCCGATAGAGTTACCCG

GAPDH forward: ACATCGCTCAGACACCATG

GAPDH reverse: TGTAGTTGAGGTCAATGAAGGG

Gapdh forward: CTTTGTCAAGCTCATTTCCTGG

Gapdh reverse: TCTTGCTCAGTGTCCTTGC

Cell transfection

BEAS-2B and A549 cells were plated in 12-well plates at 5 × 10^4 cells/well and cultured until reaching ~50% confluence. Transfection was performed using Polyplus Transfection reagent (409-10, France) with either non-targeting control siRNA or gene-specific siRNA oligonucleotides per manufacturer's protocol. Gene-specific siRNA sequences were provided by GenePharma (China) as follows:

si *CEBPB* sence: 5’- CCUCGCAGGUCAAGAGCAA(dT)(dT)-3’

si *CREB1#1* sence: 5’- CCAACAAAUGACAGUUCAA(dT)(dT)-3’

si *DDX5* sence: 5’- GCUUCGUGAAGCUAAUCAA(dT)(dT)-3’

si *RUNX1* sence: 5’- CUCAAUCGGCUUGUUGUGA(dT)(dT) -3’

si *ZEB1* sence: 5’- CCUCUCUGAAAGAACACAUUA(dT)(dT) -3’

si *ELF1* sence: 5’- CAAGGCUACUUGUCCUAAA(dT)(dT) -3’

si *SUZ12* sence: 5’- CAAUACAAGGCAACAAACU(dT)(dT)-3’

si *TAF7* sence: 5’- GGUAUUCGGUCACAGCAUA(dT)(dT)-3’

si *KLF4* sence: 5’- GCAGCUUCACCUAUCCGAU(dT)(dT)-3’

si *YY1* sence: 5’- CGACGACUACAUUGAACAA(dT)(dT)-3’

si *TCF12* sence: 5’- CAUUCAGUCCUGUCUAGUA(dT)(dT) -3’

si *CREB1#2* sence: 5’- GCAGCUCAUGCAACAUCAU(dT)(dT)-3’

si *FGFR1#2* sence: 5’- CUAUCGGACUCUCCCAUCA(dT)(dT)-3’

si *DDR1* sence: 5’- CCCUCUGCAUGAUUACUGA(dT)(dT)-3’

si *FLT3* sence: 5’- GGUGUCGAGCAGUACUCUA(dT)(dT)-3’

si *JAK1* sence: 5’- GCGAUAUAUUCCAGAAACA(dT)(dT)-3’

si *JAK2* sence: 5’- CCAUCAUACGAGAUCUUAA(dT)(dT)-3’

si *PDGFRB* sence: 5’- UUGAUCUGUAGCUGGAAGG(dT)(dT)-3’

si *FGFR1#1* sence: 5’- CUAUCGGACUCUCCCAUCA(dT)(dT)-3’

si *FGFR2* sence: 5’- CCACGGACAAAGAGAUUGA(dT)(dT)-3’

si *NTRK1* sence: 5’- GGAAGUUCAUCCAGUGUUA(dT)(dT)-3’

si *NTRK3* sence: 5’- CGAUGGUUUCAGACGCUGAAG-3’

si *VEGFR2* sence: 5’- GGAAGAUAGUGAAAUACAA (dT)(dT)-3’

Statistical Analysis

Statistical analyses were executed with GraphPad Prism 8.0. Results are expressed as mean ± SEM. Intergroup comparisons employed unpaired two-tailed Student's t-tests, while multigroup analyses utilized one-way ANOVA.

Funding: This study was supported by the Central Guidance for Local Science and Technology Development Fund Project (No. 2024ZY01047 to Dr. Luo) and the National Natural Science Foundation of China (No. 82173893 to Dr. Luo).

Supplementary Material

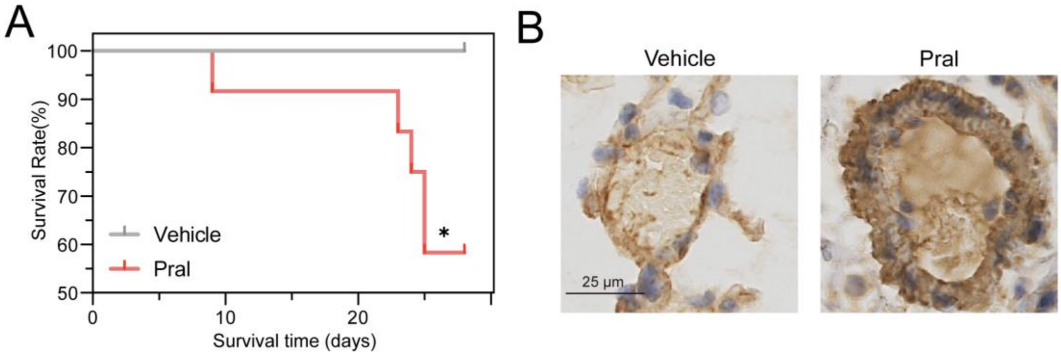

Figure S1. Pral-associated fatal interstitial lung disease.

(A) Survival curve of Vehicle- and Pral-treated (100 mg/kg/d) mice over 4 weeks. (B) Representative IHC images demonstrating pulmonary vascular remodeling in lung tissues from Vehicle- and Pral-treated mice. Statistical significance for survival was determined using the Log-rank (Mantel-Cox) test. Significance thresholds: * ($P < 0.05$).

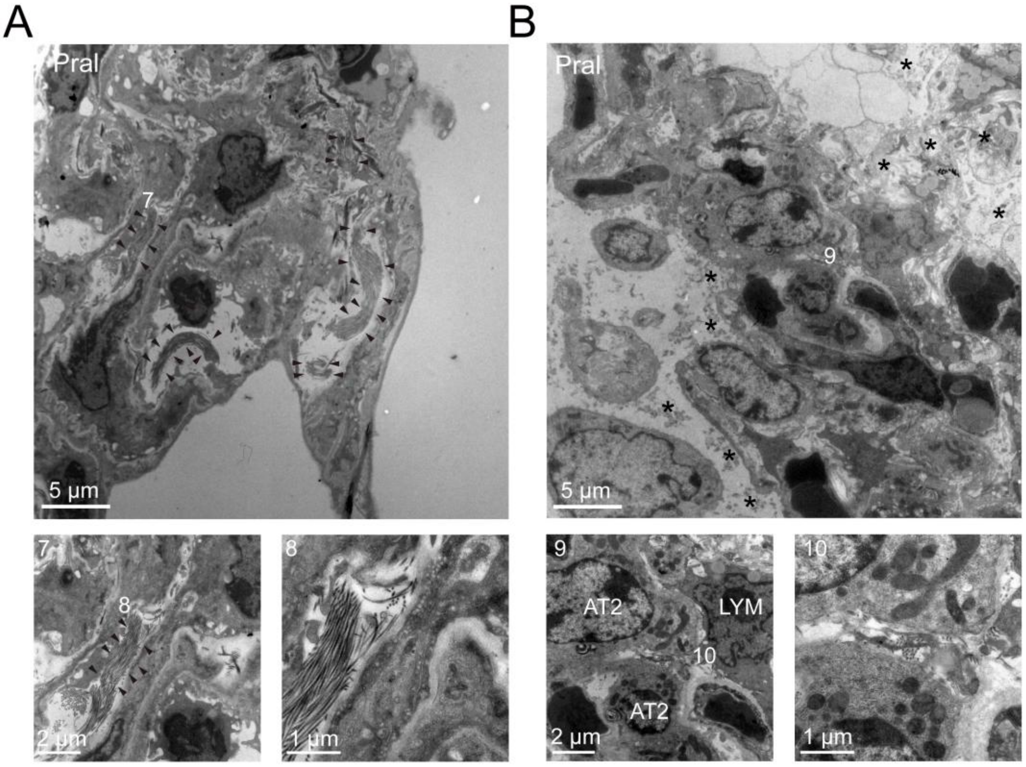

Figure S2. Electron microscopy of Pral-triggered ultrastructural changes in the Disrupted pulmonary epithelial TJ.

(A-B) Damaged pulmonary epithelial cells after Pral treatment. (A: 7-8, Fibrotic deposition, B: 9-10, Inflammatory cell infiltration) AT1: Type I alveolar epithelial cell, AT2: Type II alveolar epithelial cell, LYM: lymphocyte. Black arrows indicate fibrosis, asterisks indicate evident parenchymal edema.

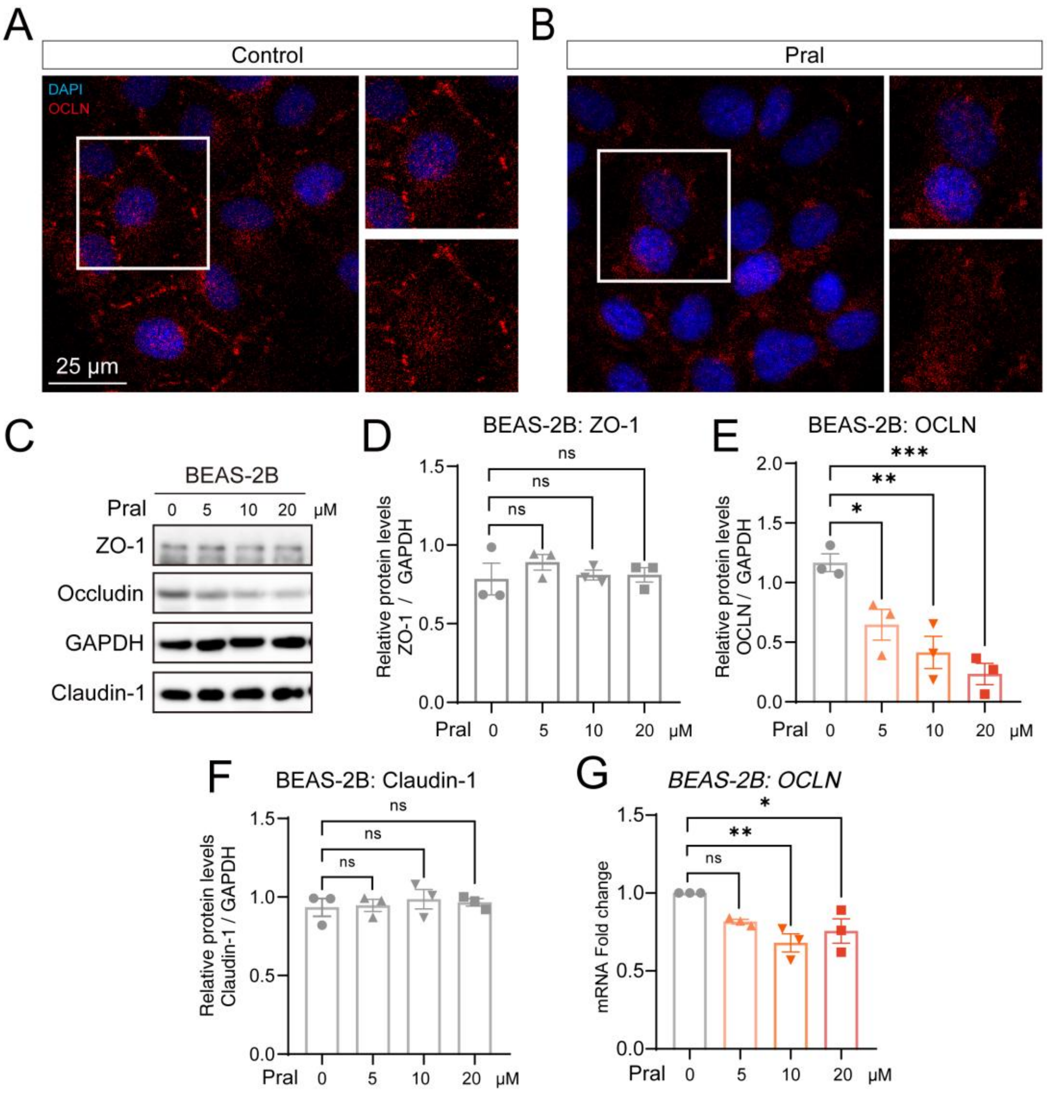

Figure S3. Pral disrupts pulmonary epithelium TJ by suppressing OCLN expression.

(A-B) Immunofluorescence (IF) staining of OCLN (red) in pulmonary epithelial cells BEAS-2B. (A) Control group. (B) Pral-treated group. (C-F) Western blot analysis of TJ proteins in BEAS-2B cells. (C) Representative blots of ZO-1, OCLN, and Claudin-1. (D-F) Quantification of protein levels for ZO-1 (D), OCLN (E), and Claudin-1 (F). (G) qRT-PCR analysis of OCLN mRNA expression in BEAS-2B cells post-Pral treatment. Statistical comparisons between multigroup analyses employed one-way ANOVA. Significance thresholds: ns (not significant), * ($P < 0.05$), ** ($P < 0.01$), *** ($P < 0.001$).

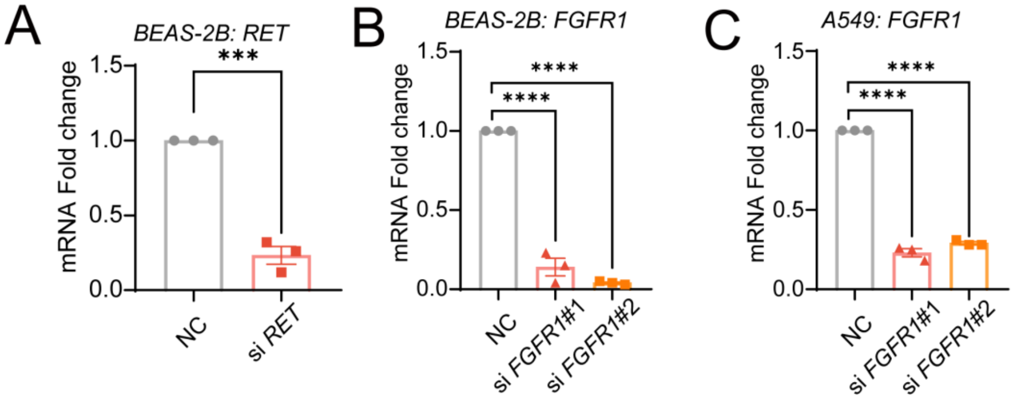

Figure S4. Pral reduces OCLN expression through FGFR1 inhibition.

(A) RET knockdown efficiency in BEAS-2B cells transfected with siRET . (B) *FGFR1* knockdown efficiency in BEAS-2B cells transfected with si*FGFR1*. (C) *FGFR1* knockdown efficiency in A549 cells transfected with si*FGFR1*.

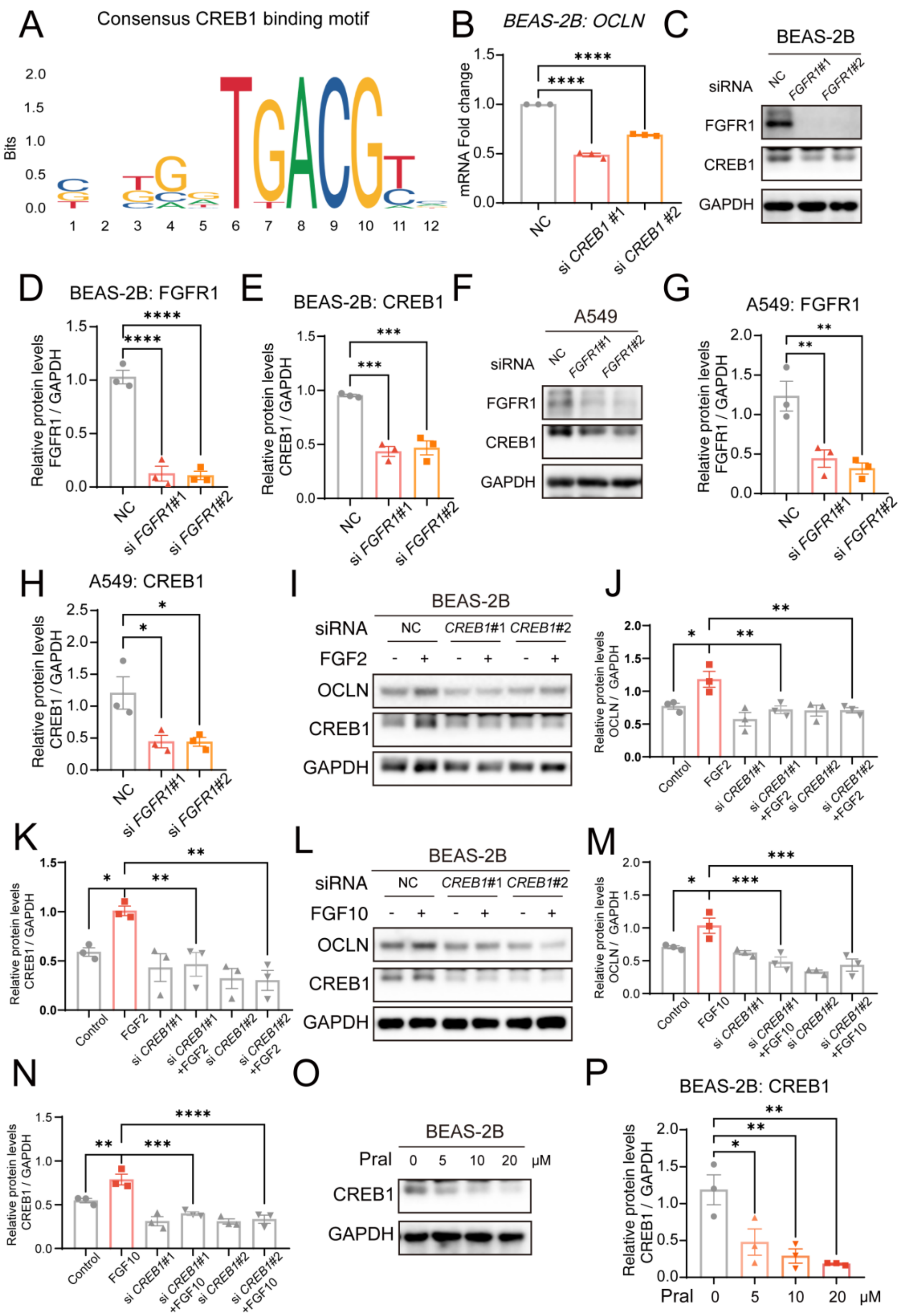

Figure S5. FGFR1 inhibition reduces OCLN transcription via CREB1 suppression.

(A) Schematic of CREB1 binding sites on OCLN promoter predicted by JASPAR database. (B) qRT-PCR analysis of OCLN mRNA in CREB1-silenced BEAS-2B cells.

(C-E) Western blot analysis in *FGFR1*-silenced BEAS-2B cells. (C) Representative blots of FGFR1 and CREB1. (D) Quantification of FGFR1.(E) Quantification of CREB1. (F-H) Western blot analysis in *FGFR1*-silenced A549 cells. (F) Representative blots. (G) Quantification of FGFR1. (H) Quantification of CREB1. (I-K) Western blot analysis in BEAS-2B cells stimulated with FGF2 (+/- si-*CREB1*). (I) Representative blots; (J-K) Quantification of OCLN and CREB1 levels. (L-N) Western blot analysis in BEAS-2B cells stimulated with FGF10 (+/- si-*CREB1*). (L) Representative blots; (M-N) Quantification of OCLN and CREB1 levels. (O-P) Western blot analysis and quantification of CREB1 expression in Pral-treated BEAS-2B cells.

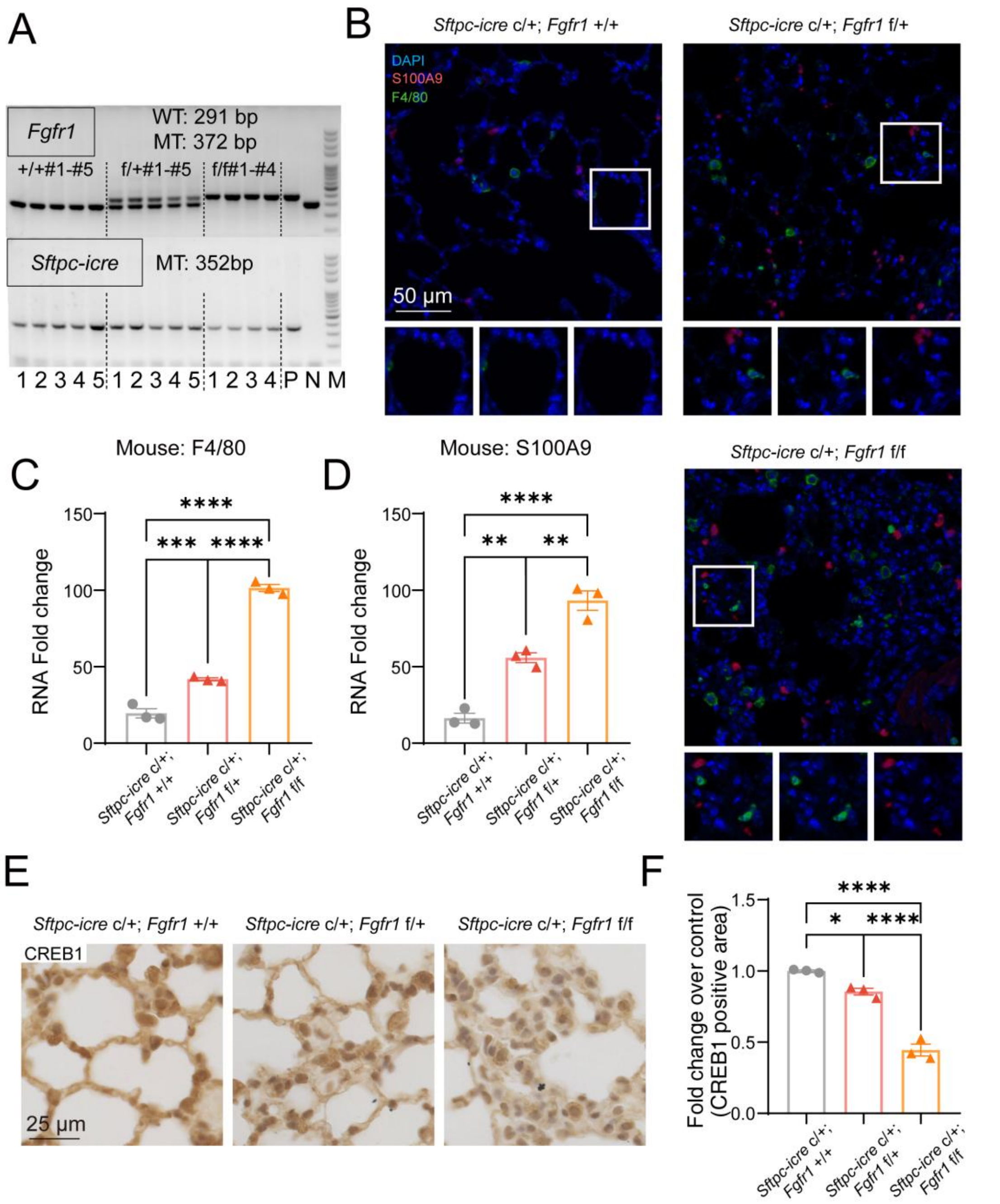

Figure S6. Lung Epithelial-Specific *Fgfr1* Knockout Induces Inflammatory Infiltration and Reduces CREB1 Expression. (A) Genotyping results confirming lung epithelial-specific *Fgfr1* knockdown/knockout (KD/KO) in mice. (B-D) Multiplex immunohistochemistry (mIHC) and quantification of immune cell infiltration in lung tissues from control and *Fgfr1* KD/KO mice. (B) Representative mIHC images of F4/80^{+} macrophages (green) and S100A9^{+} neutrophils (red). (C-

D) Quantification of average fluorescence intensity for F4/80 (C) and S100A9 (D). (E-F) IHC and quantification of CREB1 expression in lung tissues. (E) Representative IHC images of CREB1 in control and *Fgfr1* KD/KO mice. (F) Quantification of CREB1 IHC-positive area. Statistical comparisons between multigroup analyses employed one-way ANOVA. Significance thresholds: ns (not significant), * (P < 0.05), ** (P < 0.01), *** (P < 0.001), **** (P < 0.0001).

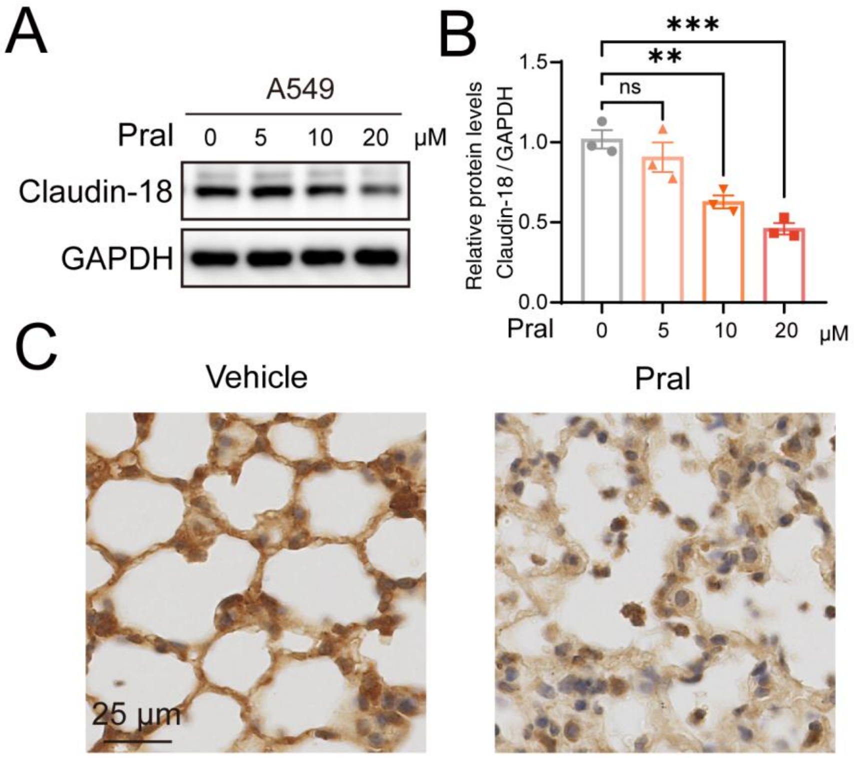

Figure S7: Pralsetinib suppresses Claudin-18 expression in lung epithelium and tissues. (A-B) Western blot analysis of Claudin-18 expression in A549 cells following Pral treatment. (A) Representative protein bands for Claudin-18 and GAPDH; (B) Quantitative analysis of Claudin-18 protein levels normalized to GAPDH. (C) Representative immunohistochemistry (IHC) images demonstrating the expression and distribution of Claudin-18 in mouse lung tissues from the Vehicle and Pral-treated

groups. Data are presented as mean ± SEM from at least three independent biological replicates. Statistical significance was determined using one-way ANOVA. ns (not significant), **: $P < 0.01$; ***: $P < 0.001$.
